# Sinistrals are rarely “right”: evidence from tool-affordance processing in visual half-field paradigms

**DOI:** 10.3389/fnhum.2015.00166

**Published:** 2015-03-27

**Authors:** Bartosz Michałowski, Gregory Króliczak

**Affiliations:** ^1^Action and Cognition Laboratory, Department of Social Sciences, Institute of Psychology, Adam Mickiewicz University in PoznańPoznań, Poland; ^2^Faculty of English, Adam Mickiewicz University in PoznańPoznań, Poland

**Keywords:** laterality, categorization, priming, tools, left-handers, visual half field

## Abstract

Although current neuroscience and behavioral studies provide substantial understanding of tool representations (e.g., the processing of tool-related affordances) in the human brain, most of this knowledge is limited to right-handed individuals with typical organization of cognitive and manual skills. Therefore, any insights from these lines of research may be of little value in rehabilitation of patients with atypical laterality of praxis and/or hand dominance. To fill this gap, we tested perceptual processing of man-made objects in 18 healthy left-handers who were likely to show greater incidence of right-sided or bilateral (atypical) lateralization of functions. In the two experiments reported here, participants performed a tool vs. non-tool categorization task. In Experiment 1, target and distracter objects were presented for 200 ms in the left (LVF) or right (RVF) visual field, followed by 200 ms masks. In Experiment 2, the centrally presented targets were preceded by masked primes of 35 ms duration, again presented in the LVF or RVF. Based on results from both studies, i.e., response times (RTs) to correctly discriminated stimuli irrespective of their category, participants were divided into two groups showing privileged processing in either left (*N* = 9) or right (*N* = 9) visual field. In Experiment 1, only individuals with RVF advantage showed significantly faster categorization of tools in their dominant visual field, whereas those with LVF advantage revealed merely a trend toward such an effect. In Experiment 2, when targets were preceded by identical primes, the “atypical” group showed significantly facilitated categorization of non-tools, whereas the “typical” group demonstrated a trend toward faster categorization of tools. These results indicate that in subjects with atypically organized cognitive skills, tool-related processes are not just mirror reversed. Thus, our outcomes call for particular caution in neurorehabilitation directed at left-handed individuals.

## Introduction

In typical right-handed individuals, the processing of information about tools takes place primarily in their left hemispheres (for reviews, see Johnson-Frey, 2004; Lewis, 2006; see also Orban and Caruana, 2014; Vingerhoets, 2014). Interestingly, in the case of tool-related manual skills, the engagement of left-lateralized processes is apparent even when an interaction with a tool is performed with the non-dominant (left) hand (e.g., Johnson-Frey et al., 2005; Króliczak and Frey, 2009). Whether or not the neural underpinning of tool-use skills in left-handed individuals (sinistrals) exhibits the same asymmetry is currently debated (Vingerhoets et al., 2012; Goldenberg, 2013). Surprisingly, this discussion takes place in the absence of systematic research on representations underlying perceptual processing of tools and other man-made objects in this often-discarded (or rather underrepresented in scientific research) population (for a review on this and other topics, see Willems et al., 2014).

Although both neuropsychological (Goldenberg, 2013) and neuroimaging (Vingerhoets et al., 2012) data from sinistrals, as compared to dextrals (right-handers), point to a less asymmetric organization of functions, it is yet to be determined if such an effect is due to a tendency for all left-handers to have their brains more symmetrically organized or due to a rather higher incidence of atypical representation of functions introducing bias in the group data from this population (for a discussion, see Króliczak, 2013a). Indeed, this is quite likely given the evidence showing that up to 30% of left-handed individuals demonstrate atypical—i.e., bilateral or right-sided—organization of cognitive skills such as language (Knecht et al., 2000), praxis, or both (Króliczak et al., 2011; Vingerhoets et al., 2013; see also Meador et al., 1999). If such a pattern was a reflection of a more general organization of functions in their brains, one would predict that left-handers with atypically organized higher-order manual skills would also exhibit atypical laterality of processing underlying the categorization of tools (cf. Ochipa et al., 1989). Testing for this possibility is paramount because, in the long run, it has a clear potential to reveal handedness-independent interrelations of cognitive functions in the brain, whether typical or not.

The easiest and arguably most effective way of addressing this issue is the use of a visual half-field (VHF) paradigm, which is a reliable measure of hemispheric dominance of functions when used properly (Hunter and Brysbaert, 2008; Verma and Brysbaert, 2011; see also: Garcea et al., 2012; Helon and Króliczak, 2014). In the majority of studies that were related to tool processing, however, the issue of typical and atypical representation of this cognitive skill has never been directly addressed (cf. Verma et al., 2013). Notably, one of the first reports to investigate the laterality of tool representations with the use of VHF paradigm was a paper by Verma and Brysbaert (2011), who tested their right-handed participants on a categorization task with bilaterally presented man-made objects (tools, and non-tools). Yet, the sample they used did not allow them to pose a question of typical vs. atypical processing of the tool category. Therefore, in line with previous studies that drew their conclusions only from right-handers (for a review, see: Lewis, 2006), when averaging across tests and participants, the mere effect they observed was some right visual-field (RVF) advantage for the categorization of man-made objects, including tools. A somewhat stronger effect was observed in a study that utilized a different VHF test, i.e., a lateralized masked priming paradigm, by Garcea et al. (2012), in which participants categorized centrally shown pictures of tools or animals preceded by laterally presented identical or scrambled primes. The priming effect they observed only for tools again indicated the RVF advantage for tool categorization. Given that the majority of subjects involved were right-handed, a chance of finding a subset of individuals with atypically represented tool-processing skills was neither high, nor addressed.

In this study, we investigated the processing of tool-related information exclusively in left-handers, a population offering a higher incidence of individuals with atypically lateralized functions (e.g., Króliczak et al., 2011). We wanted to ensure that the to-be-obtained results would specifically concern tools as a unique type of human artifacts. Therefore, the **tool category**—for which the object concept is linked not only to the relevant functional properties of that object *type* but also to a set of invariant, use-related properties or *stable affordances* (e.g., the type of grip required when manipulating the tool in accordance with its function, Borghi and Riggio, 2009; see also Tucker and Ellis, 2004; Bub et al., 2015; cf. the *micro-affordance* concept by Ellis and Tucker, 2000) that trigger the relevant representations of manual skills (e.g., Vainio et al., 2008; Bub et al., 2013)—was contrasted with **other man-made objects** (i.e., non-tools), a wider category of human artifacts for which *manipulability* is no longer that important but some function is still present. Specifically, we tested: (1) whether or not a difference in visual processing of tools vs. other man-made objects would be observed in accuracy and response times (RTs) in two disparate paradigms utilizing VHF presentations, (2) whether or not the potential left-right asymmetry demonstrated in such experiments would be homogenous across left-handers or, conversely, would allow us to divide the group into two different samples showing advantage for one or the other visual field, and (3) whether or not this pattern of performance would be consistent within a group across the selected behavioral tasks.

We hypothesized that a VHF advantage would be present only for the processing of pictures of tools. Specifically, we expected that our left-handed participants would split into two groups, one showing left visual field (LVF) advantage for tool processing, and the other demonstrating the typical, RVF advantage. Finally, we predicted that the processing of non-tools would be unaffected by the side of presentation (Experiment 1), or the side in which the prime appeared (Experiment 2), irrespective of the group.

## Experiments

Although the order of the two experiments described here—one with laterally presented targets (in either VHF), and one with laterally presented primes (in either VHF)—was counterbalanced across participants, for simplicity we will nevertheless refer to the presentation of target objects in VHFs as Experiment 1, and to the presentation of primes in VHFs as Experiment 2. Both experiments were run in *Action and Cognition Laboratory* in the Institute of Psychology at Adam Mickiewicz University in Poznań, Poland. The study was approved by the local Ethics Committee for Research Involving Human Subjects and was carried out in accordance with the principles of the Helsinki 1964 Declaration.

Eighteen healthy left-handed volunteers (undergraduate or postgraduate students, 9 women, mean age = 23.3, *SD* = 3.7) took part in Experiment 1 and Experiment 2, and both experiments were undertaken with the understanding and written consent of each participant. All subjects had normal or corrected-to-normal visual acuity and, as established by the revised version of the Edinburgh Handedness Inventory (Oldfield, 1971; Dragovic, 2004), were strongly left-handed (mean laterality quotient = −83.9, *SD* = 22.1).

Before conducting any analyses we examined whether or not there are any atypical cases among our participants based on their responses to all stimuli presented to the left or right visual field. Consequently, two laterality indices (LI_1_ for Experiment 1 and LI_2_ for Experiment 2) were calculated for each individual in the following way: LI_1_ = [(L_1_ − R_1_)/(L_1_ + R_1_)] × 100, where L_1_ and R_1_ represent RTs for targets (tools and non-tools) presented in the left (L_1_) or right (R_1_) VHF, respectively, and LI_2_ = [(L_2_ − R_2_)/(L_2_ + R_2_)] × 100, where L_2_ and R_2_ represent RTs for targets (tools and non-tools) preceded by identity primes presented, again, in the left (L_2_) or right (R_2_) VHF. Each individual's LI_1_ and LI_2_ were then averaged to form a measure of general visual field dominance, LI_G_[LI_G_ = (LI_1_ + LI_2_)/2]. Participants with LI_G_ < 0 were classified as representing left visual-field advantage group (LVF-A, *N* = 9, 5 women), whereas those with LI_G_ > 0 were classified as representing right visual-field advantage group (RVF-A, *N* = 9, 4 women). Despite different directions of the visual field asymmetries, the groups did not differ from each other in terms of the actual strength of these asymmetries [*t*_(16)_ = 0.29, *p* = 0.76] as measured in absolute values.

## Experiment 1: categorization of target objects presented in LVF or RVF

### Methods

The design of Experiment 1 was based on that used by Verma and Brysbaert (2011) with some modifications.

#### Stimuli

The stimuli consisted of 60 line-drawings of familiar man-made objects (30 tools, 30 non-tools; the list of all pictures can be found in the Appendix 1) from the set of 400 pictures used by Cycowicz et al. (1997). They were downloaded from the website of the Cognitive Electrophysiology Laboratory (CEPL) at the New York State Psychiatric Institute and Columbia University Medical Center (http://nyspi.org/cepl/resources) with the consent of one of the authors. Half of the objects from each category (15 tools and 15 non-tools) were rotated so that the long axis of the object was deflected from the vertical by 45°, whereas objects from the other half were rotated in the same manner to obtain a deflection of 315°. All images were sized to 140 × 140 pixels.

#### Procedure

Before the experiment proper, participants were familiarized with all the stimuli. Images of tools and non-tools were presented in the middle of the screen on a white background. The name of the object was displayed below the picture; the name of the category—above it. Each slide was presented for 3000 ms to ensure proper familiarization with the category of the objects to be shown in the experimental task. Subsequently, a training session of 24 trials was administered, and it involved an equal number of randomly selected pictures from both categories.

**Figure 1:**
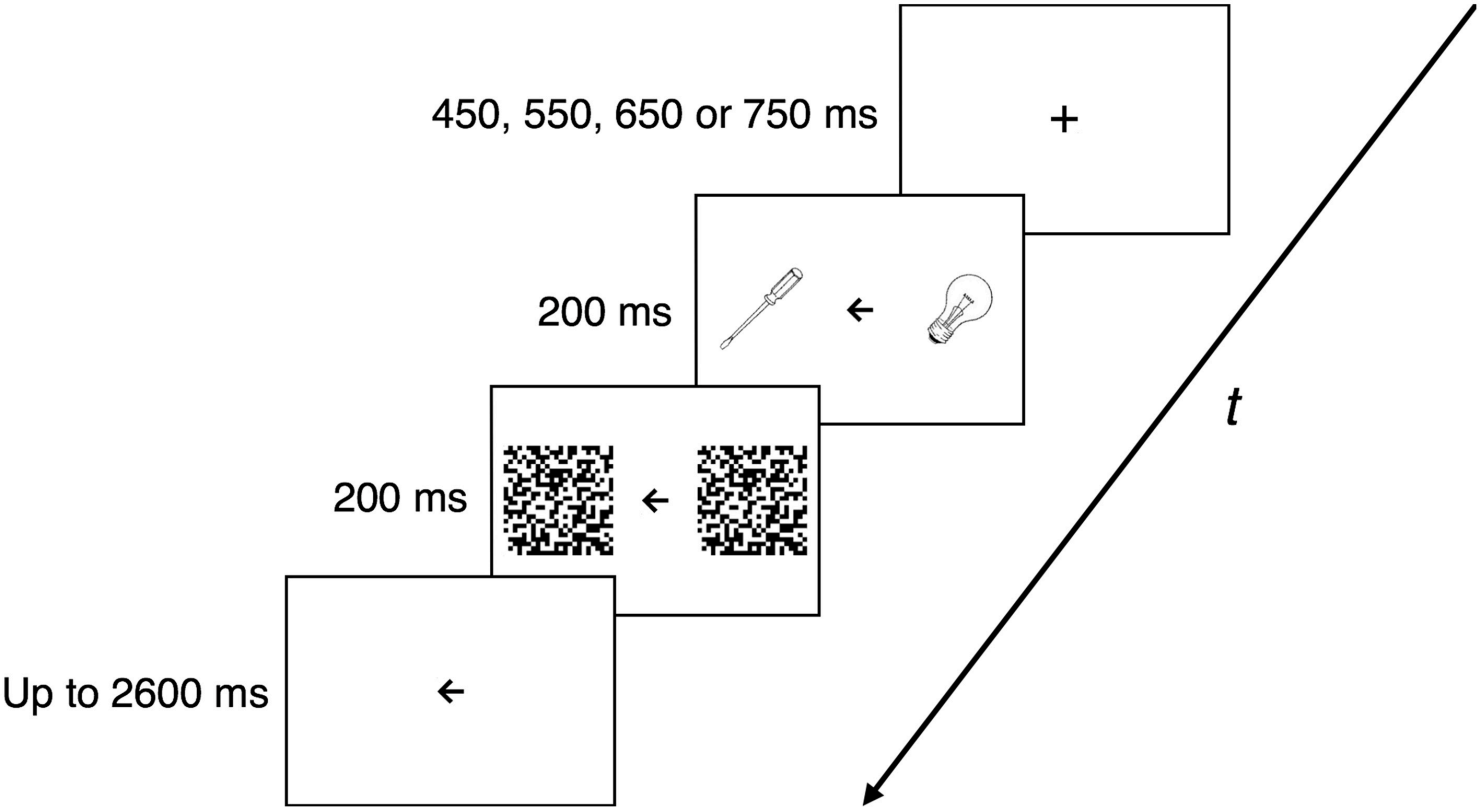
**Trial structure and timing in Experiment 1**. After a fixation point presented on a blank screen for a variable time interval (450, 550, 650, or 750 ms), two stimuli (the target and the distracter) were shown bilaterally for 200 ms, with a central arrow pointing to the location of the target. The stimuli were then covered by 200-ms masks. The arrow stayed on the screen until a participant responded or for up to 3 s of total presentation time. A 1000-ms blank screen separated successive trials.

Participants were seated in front of the screen at a viewing distance of ~57 cm. Each trial began with a central fixation cross (sized 1° of visual angle) of variable (450, 550, 650, or 750 ms) duration. Next, two images of different objects belonging to the same or different category (tool vs. non-tool) were presented in the left and right visual field (starting at 3° of visual angle from the middle of the screen; both images sized 4° of visual angle) with a central arrow (sized 1° of visual angle) pointing to the left or right. The role of the arrow was to indicate the stimulus to which attention should be paid to. After 200 ms, the images were replaced with black-and-white high-contrast pattern masks for another 200 ms. Similarly to the study by Verma and Brysbaert (2011), the task was to decide (as quickly and accurately as possible) whether the target object was a tool or non-tool. The arrow remained on the screen until the participant responded, but for no longer than 2600 ms after the disappearance of the masks. Participants were asked to respond bimanually with their index fingers when the target was a tool and with their middle fingers when the target was a non-tool. The reaction time, as measured by the first key press, and accuracy of this response were recorded by the software used for stimulus presentation. A 1000-ms blank screen was introduced between the successive trials. The trial structure is depicted in Figure 1.

The design was implemented in SuperLab ver. 4.5.2 (Cedrus®, San Pedro, CA). The stimuli were presented on a 20 inch CRT monitor with a refresh rate of 85 Hz and a resolution of 1280 × 960. “RB-730” response pad by Cedrus was used for measuring accuracy and RTs. Every participant completed two blocks of randomly presented 240 trials with a 2-min break between the blocks. Each of the 60 stimuli was presented four times in each block: twice in the LVF (with compatible or incompatible distracters) and twice in the RVF (again with compatible or incompatible distracters). Care was taken to ensure that the two images presented in every trial were randomly paired for each participant and depicted different objects.

All the collected data were analyzed with four separate repeated-measures Analyses of Variance (ANOVAs), two for RTs to correctly categorized objects and two for accuracy. In the within-subjects analyses, the factors were *target location* (LVF, RVF), *target category* (tool, non-tool), and *distracter compatibility* (compatible, incompatible). In the mixed analyses, we included an additional, between-subjects factor, i.e., *group* (LVF-A, RVF-A), in order to account for the fact that each half of our participants demonstrated the opposite overall visual field advantage. The adopted level of significance was alpha = 0.05. The required follow-up tests of simple main effects were Bonferroni corrected (marked Bf-*p*). For reaction times accompanying a correct categorization of objects, outliers greater than two standard deviations above or below the mean (calculated for each participant in each condition, 4.9% of all trials) were removed. Statistical analyses were carried out using SPSS 21.0 (SPSS Ins., Chicago, IL).

### Results

#### Within-subjects analyses

#### Recognition accuracy

We observed a clear trend toward a main effect of *target category* which just missed the adopted significance level [*F*_(1, 17)_ = 4.11, *p* = 0.06, Partial Eta Squared (_*p*_η^2^) = 0.19]. Namely, participants showed a strong tendency for more accurate categorization of non-tools than tools (average accuracy for non-tools = 84%, *SE* = 2.6% vs. tools = 76.2%, *SE* = 4.2%). The main effects of *target location* and *distracter compatibility* were not significant [*target location*: *F*_(1, 17)_ = 0.09, *p* = 0.76, _*p*_η^2^ = 0.01; *distracter compatibility*: *F*_(1, 17)_ = 0.01, *p* = 0.92, _*p*_η^2^ = 0.001]. There was also a trend toward a significant interaction between *target category* and *distracter compatibility* [*F*_(1, 17)_ = 3.76, *p* = 0.07; _*p*_η^2^ = 0.18], indicating that when a distracter was compatible with the target, non-tools were categorized with greater accuracy than tools (average accuracy for non-tools = 84.7%, *SE* = 2.6% vs. tools = 75.5%, *SE* = 4.3%, Bf-*p* = 0.06). None of the remaining interactions was statistically significant.

#### Response times (RTs) to correctly categorized objects

Neither *target location*, nor *target category* or *distracter compatibility* had a significant effect on RTs to correctly categorized stimuli [*target category*: *F*_(1, 17)_ = 0.62, *p* = 0.44, _*p*_η^2^ = 0.04; *target category*: *F*_(1, 17)_ = 0.001, *p* = 0.97, _*p*_η^2^ = 0.00; *distracter compatibility: F*_(1, 17)_ = 2.36, *p* = 0.14, _*p*_η^2^ = 0.12]. None of the interactions reached the significance threshold. The mean RTs and average accuracy for all the conditions are listed in Table 1.

**Table 1:** **Targets in VHFs - Experiment 1**.

**Trial type**			**Response time (ms)**	**St. error**	**Accuracy (%)**	**St. error**	***N***
LVF	Tool	Compatible distracter	845	47	75.5	3.9	18
		Incompatible distracter	871	52	76.9	4.3	18
	Non-tool	Compatible distracter	840	50	84.1	2.8	18
		Incompatible distracter	862	51	83.3	2.6	18
RVF	Tool	Compatible distracter	846	48	75.5	4.7	18
		Incompatible distracter	844	53	76.9	4.2	18
	Non-tool	Compatible distracter	847	56	85.4	2.6	18
		Incompatible distracter	856	46	83.1	3.0	18

*Target location (Left Visual Field, LVF; Right Visual Field, RVF), target category (tool, non-tool), distracter compatibility (compatible, incompatible) with their mean response times (ms), accuracy (%), and their standard errors of the means, for Experiment 1 with targets presented in either LVF or RVF are listed*.

#### Between-subjects (mixed) analyses

#### Recognition accuracy

No significant difference in average accuracy was found between the LVF-A and RVF-A group [*t*_(16)_ = 0.72, *p* = 0.48]. In addition to a trend toward a significant main effect of *target category* [*F*_(1, 16)_ = 4.27, *p =* 0.06, _*p*_η^2^ = 0.21] and a trend toward a significant interaction between *target category* and *distracter compatibility* [*F*_(1, 17)_ = 3.95, *p* = 0.06, _*p*_η^2^ = 0.20], that were both reported above, there was now also a significant interaction between *group, target location* and *distracter compatibility* [*F*_(1, 16)_ = 5.84, *p* < 0.05, _*p*_η^2^ = 0.27], but none of the *post-hoc* tests survived the Bonferroni correction.

#### Response times (RTs) to correctly categorized objects

**Figure 2:**
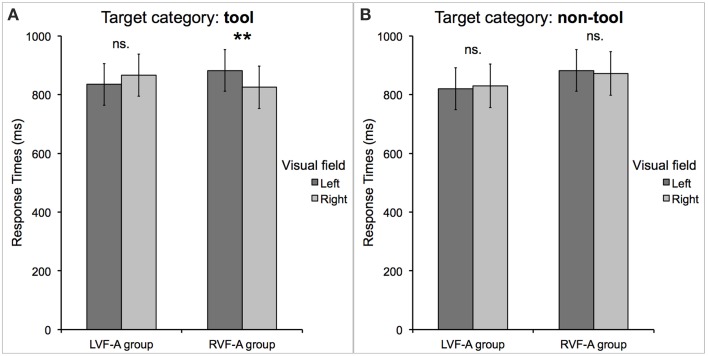
**Response times to correctly categorized (A) tools and (B) non-tools displayed as a function of the group (representing left or right visual field advantage) and the attended visual field in which the target occurred**. The only significant effect was observed in the performance of the *typical*, right visual field advantage group (RVF-A), who categorized tools significantly faster when they were presented to the right of the fixation point. The *atypical*, left visual field advantage group (LVF-A) showed only a trend toward a similar effect for tools presented to the left. Asterisks indicate a difference with Bonferroni-corrected *p*-value of 0.01 (^**^).

Again, there was no significant difference in the mean RTs between the LVF-A and RVF-A group [*t*_(16)_ = 0.28, *p* = 0.78]. Importantly, we found a significant interaction between *group, target location* and *target category* [*F*_(1, 16)_ = 6.18, *p* < 0.05, _*p*_η^2^ = 0.28]. Namely, participants in the RVF-A group showed significantly faster categorization of tools presented in the RVF as compared to the LVF (mean RT in the RVF = 825 ms, *SE* = 72 ms vs. LVF = 882 ms, *SE* = 71 ms; Bf-*p* < 0.01). In contrast, participants in the LVF-A group showed a different pattern: although their responses were faster to tools correctly categorized in the LVF as compared to the RVF (mean RT for the LVF = 835 ms, *SE* = 71 ms vs. RVF = 866 ms, *SE* = 72 ms), this tendency did not reach the significance threshold after the Bonferroni correction (Bf-*p* = 0.13). Neither group showed any significant VHF dominance for non-tool categorization (LVF-A group: Bf-*p* = 1.00; RVF-A group: Bf-*p* = 1.00). These effects are shown in Figure 2.

### Discussion of experiment 1

The paradigm used in Experiment 1 provides a unique approach to investigating the laterality of mechanisms involved in the categorization (or even recognition) of stimuli of different kinds. Namely, a required cognitive decision is made on the basis of a target stimulus presented laterally, i.e., appearing exclusively in one of the two VHFs (though accompanied by a non-target on the opposite side), and thus projected only to the contralateral hemisphere. Therefore, any preferential stimulus *processing* observed in the left or right VHF indicates that the most relevant mechanisms, e.g., here: for the extraction of tool-specific affordances, are predominantly lateralized to the right or left hemisphere, respectively.

In light of the above assumptions, the lack of the main effect of *target location* (or an interaction between *target location* and *target category*) observed both in accuracy and RTs to correctly categorized stimuli in the within-subjects analyses could be regarded as quite surprising. This is no longer the case, however, when one realizes that the left-handed participants we studied clearly represented two disparate groups, each demonstrating visual field advantage on opposite sides. After taking this distinctive attribute into account, i.e., by introducing into our analyses the *group* factor—which, notably, was independent of the task (or experiment) and stimulus type, we found different patterns of RTs to correctly categorized stimuli.

The right visual field advantage for the categorization of tools observed for RTs in the “typical” (RVF-A) group is consistent with a well-established role of the left hemisphere in encoding and retrieval of visual representations of tools (e.g., Grafton et al., 1997; Perani et al., 1999; Verma and Brysbaert, 2011; Garcea et al., 2012; or tool-use skills, e.g., Helon and Króliczak, 2014; cf. Króliczak, 2013b). A trend toward the LVF advantage observed in the “atypical” (LVF-A) group for tool categorization reveals another important finding, namely that the strength of the involvement of the right-hemisphere mechanisms in processing of human artifacts—and particularly tools—varies substantially across this group of individuals. Indeed, among the subjects with the putative atypical organization of object processing (and perhaps other cognitive skills) there were two participants who despite showing a clear general LVF advantage (irrespective of the task and stimulus kind) did not reveal such an effect for tools. Therefore, it should be emphasized at this point that such a result is not an artifact of the *grouping method* adopted in our study. A very similar pattern of outcomes has been reported by Verma et al. (2013) in a VHF study on symmetry detection wherein participants with known atypical hemispheric dominance for speech demonstrated greater variability in the studied task, with only about half of them showing LVF advantage for the processing of symmetrical shapes.

The faster categorization of tools observed in the RVF-A group in the dominant VHF and a similar (though much weaker) effect observed in the LVF-A group, as opposed to no comparable effect of any kind for non-tool stimuli, is also consistent with the idea that information about tools, in contrast to other objects (e.g., animals, houses, or graspable shapes with no function), is processed in the brain in a unique way (e.g., Chao et al., 1999; Chao and Martin, 2000; Creem-Regehr and Lee, 2005). In fact, nearly all studies on tasks involving tools in typical (usually right-handed) individuals point to the left hemisphere as the seat of their representations (including their concepts and the relevant manual skills). It is also worth mentioning that our finding of no visual field asymmetry in the accuracy or speed of categorization for non-tools is furthermore in line with numerous neuroimaging and behavioral studies, too (e.g., Biederman and Cooper, 1991; Proverbio et al., 2011; Verma and Brysbaert, 2015). These reports clearly indicate that the representations (or perhaps the mechanisms involved in categorization and/or recognition) of non-manipulable objects are organized more bilaterally. That is, none of the hemispheres seems to be preferentially involved in their encoding and retrieval.

Notably, the lack of preferential involvement of any hemispheres for non-tools did not prevent our participants from being more accurate in their processing (there was at least a clear trend toward greater accuracy in the categorization of non-tools as compared to tools, irrespective of distracter's category). Although this finding may just indicate that our sample was basically more familiar with non-tool objects included in this study (and the presence of compatible distracters seemed to facilitate their categorization even more), this result goes against a hypothesis that a greater expertise with a given category of objects may be accompanied by a more localized and/or lateralized processing (such an argument seems to be tacitly assumed in many studies on tool representations).

But is it really a specific mechanism rather than a more general processing stream that was tackled with the use of the VHF paradigm that we adopted in Experiment 1? Alternatively, can any results obtained with such an approach really tell us anything about the inner organization of the processes that are involved in the task of interest? In our opinion, some light on this issue can be shed by using the laterally-presented objects as primes to the centrally-displayed targets requiring subsequent categorization. This is exactly what has been done in Experiment 2.

## Experiment 2: categorization of objects preceded by primes presented in either LVF or RVF

### Methods

The design of Experiment 2 was based on that used by Garcea et al. (2012) with some modifications.

#### Stimuli

The stimuli consisted of 60 gray-scaled pictures of familiar man-made objects (30 tools, 30 non-tools, the list of all pictures can be found in Appendix 1). As in Experiment 1, half of the objects from each category (15 tools and 15 non-tools) were rotated so that the long axis of the object was deflected from vertical by 45°, whereas objects from the other half were rotated in the same manner to obtain a deflection of 315°. Seventy percent of additive noise was overlaid on all the pictures (for a rationale of this manipulation, see Garcea et al., 2012). All images were sized to 174 × 174 pixels.

#### Procedure

At the beginning of the experiment participants were familiarized with the stimuli in the same manner as in Experiment 1. They also took part in a training session of 24 trials, which again involved an equal number of randomly selected pictures from both categories.

**Figure 3:**
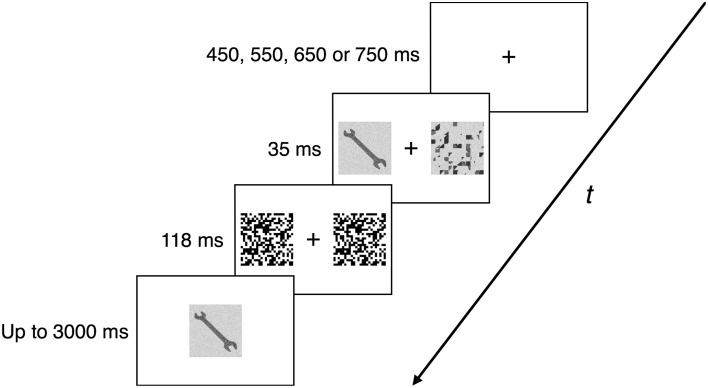
**Trial structure and timing in Experiment 2**. After a fixation point presented on a blank screen for a variable time interval (450, 550, 650, or 750 ms), the priming stimulus (identical or scrambled version of the target) was shown either on the left or right for 35 ms, with an accompanying scrambled image presented on the opposite side. Both stimuli were then covered by 118-ms masks. Next, the target image was presented centrally and stayed on the screen until a participant responded or for up to 3000 ms. A 1000-ms blank screen separated successive trials.

Each trial began with a central fixation cross (sized 1° of visual angle) of variable (450, 550, 650, or 750 ms) duration. Next, a prime (a tool or a non-tool) was presented in the left or right visual field (starting 3° of visual angle from the middle of the screen; 5° of visual angle in size). In the identity condition, the prime was same as (i.e., identical with) the to-be-seen target, while in the scrambled condition it was a scrambled version of the to-be-seen target. In both conditions, in the opposite visual field, the prime was accompanied by a scrambled version of a different image from the same category (again, starting 3° of visual angle from the middle of the screen; 5° of visual angle in size). After 35 ms, the prime and the accompanying image were immediately replaced with black-and-white high-contrast pattern masks of the same size for 118 ms. Then, the target image (a tool or a non-tool) was presented centrally and remained on the screen until the participant made a response, but for no longer than for 3000 ms. The task was to decide (as quickly and accurately as possible) whether the target was a tool or non-tool. Similarly to Experiment 1, participants responded bimanually with their index fingers if the target was a tool and with their middle fingers if the target was a non-tool. The time of the first key press and the correctness of the response were recorded. A 1000-ms blank screen was introduced between the successive trials. The trial structure is depicted in Figure 3.

The technical equipment and software used was identical to Experiment 1. Every participant completed two blocks of randomly presented 240 trials with ~2 min break between the blocks. Each of the 60 stimuli was presented four times in each block: twice in the LVF and twice in the RVF, twice in the identity condition and twice in the scrambled condition. Care was taken to ensure that the prime and the accompanying image presented in every trial depicted different objects of the same category (a tool or non-tool), randomly paired for each participant.

Similarly to Experiment 1, the collected data were analyzed with four separate repeated-measures ANOVAs, two for RTs to correctly categorized objects and two for accuracy. The within-subject factors were *prime location* (left, right), *target category* (tool, non-tool), and *prime condition* (identical, scrambled). The between-subjects factor was group (LVF-A, RVF-A). The adopted level of significance was alpha = 0.05 and, if necessary, *post-hoc* tests were Bonferroni corrected (Bf-*p*). For RTs to correctly categorized objects, outliers greater than two standard deviations above or below the mean were removed (4.8% of all trials).

### Results

#### Within-subjects analyses

#### Recognition accuracy

There was a significant main effect of *target category* [*F*_(1, 17)_ = 11.84, *p* < 0.01, _*p*_η^2^ = 0.41] such that participants categorized non-tools with greater accuracy than tools (average accuracy for non-tools = 95.9%, *SE* = 0.9% vs. tools = 88.6%, *SE* = 1.8%). There was no main effect of *prime location* [*F*_(1, 17)_ = 0.54, *p* = 0.47, _*p*_η^2^ = 0.03] or *prime condition* [*F*_(1, 17)_ = 1.66, *p* = 0.21, _*p*_η^2^ = 0.09]. There was also a significant interaction between *target category* and *prime condition* [*F*_(1, 17)_ = 6.10, *p* < 0.05, _*p*_η^2^ = 0.26], but the effect of tools being easier to categorize when identical primes instead of scrambled primes were presented just missed the significance threshold (Bf-*p* = 0.07). No further significant effects were found.

#### Response times (RTs) to correctly categorized objects

We found a significant main effect of *prime condition* [*F*_(1, 17)_ = 15.55, *p* = 0.001, _*p*_η^2^ = 0.48], indicating shorter reaction times to targets preceded by identical as opposed to scrambled primes (mean RT for identical primes = 611 ms, *SE* = 27 ms vs. scrambled primes = 628 ms, *SE* = 27 ms). There was no main effect of *prime location* [*F*_(1, 17)_ = 0.48, *p* = 0.50, _*p*_η^2^ = 0.03] or *target category* [*F*_(1, 17)_ = 0.91, *p* = 0.35, _*p*_η^2^ = 0.05]. Moreover, interactions between *prime location* and *target category, prime condition* and *target category, prime location* and *prime condition*, as well as a three-way interaction, were not significant.

#### Between-subjects (mixed) analyses

#### Recognition accuracy

Participants in the RVF-A group categorized stimuli with greater accuracy than subjects in the LVF-A group [average accuracy in the RVF-A group = 94.2%, *SE* = 1.0% vs. LVF-A group = 90.3%, *SE* = 1.4%; *t*_(16)_ = 2.24, *p* < 0.05]. We found the previously described significant main effect of *target category* [*F*_(1, 16)_ = 14.34, *p* < 0.01, _*p*_η^2^ = 0.47] and the significant interaction between *target category* and *prime condition* [*F*_(1, 16)_ = 5.90, *p* < 0.05, _*p*_η^2^ = 0.27], which showed that only in the case of tools, greater accuracy in categorization was associated with identical rather than scrambled primes (average accuracy for identical primes = 89.2%, *SE* = 1.6% vs. scrambled primes = 88%, *SE* = 1.6%; Bf-*p* = 0.05). There was also a significant interaction between *group* and *target category* [*F*_(1, 16)_ = 4.59, *p* < 0.05, _*p*_η^2^ = 0.22], showing that only the LVF-A group categorized non-tools with greater accuracy than tools (average accuracy for non-tools: 95.9%, *SE* = 1.3% vs. tools: 84.6%, *SE* = 2.2%; Bf-*p* < 0.01). A significant interaction between *group* and *prime condition* [*F*_(1, 16)_ = 5.64, *p* < 0.05, _*p*_η^2^ = 0.26] moreover indicated that only the LVF-A group categorized stimuli with greater accuracy but only when they were preceded by identical primes, as compared to scrambled primes (average accuracy for identical primes: 90.8%, *SE* = 1.2% vs. scrambled primes = 89.7%, *SE* = 1.3%; Bf-*p* < 0.05).

#### Response times (RTs) to correctly categorized objects

**Figure 4:**
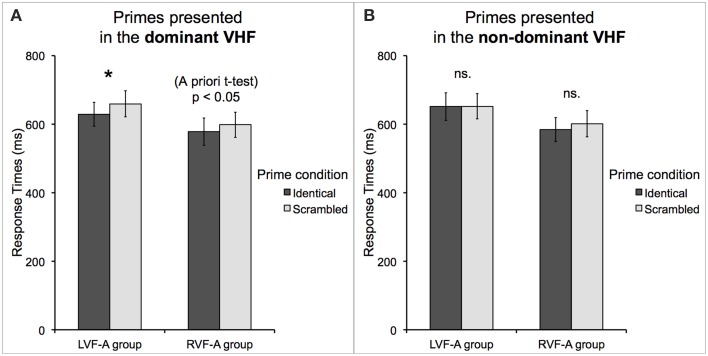
**Response times to correctly categorized objects of any category (tools and non-tools) preceded by primes presented in the (A) dominant and (B) non-dominant visual fields displayed as a function of the group (representing left or right visual field advantage) and prime type**. Regardless of object category, whereas the *atypical*, left visual field advantage group (LVF-A) showed a priming effect in its dominant (left) visual field, the *typical*, right visual field advantage group (RVF-A) demonstrated only weak priming in its dominant (right) visual field. Asterisk indicates a difference with Bonferroni-corrected *p*-value of 0.05 (^*^).

**Figure 5:**
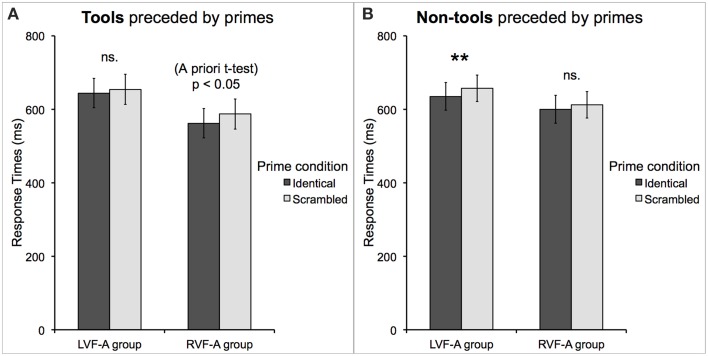
**Response times to correctly categorized (A) tools and (B) non-tools displayed as a function of the group (representing left or right visual field advantage) and prime type**. The *atypical*, left visual field advantage group demonstrated a strong priming effect only for non-tool categorization, but now irrespective of the prime side, whereas the *typical*, right visual field advantage group showed both weaker priming effect and only for the categorization of tools. Asterisks indicate a difference with Bonferroni-corrected *p*-value of 0.01 (^**^).

LVF-A group and RVF-A group did not differ significantly in the mean RTs [*t*_(16)_ = 1.09, *p* = 0.29]. As above, we found a significant main effect of *prime condition* [*F*_(1, 16)_ = 14.71, *p* = 0.001, _*p*_η^2^ = 0.48] such that targets were categorized faster when preceded by identical primes as compared to scrambled primes, and a new significant interaction between *prime location* and *prime condition* [*F*_(1, 16)_ = 4.35, *p* = 0.05, _*p*_η^2^ = 0.21] such that only in the case of left-sided priming, identical primes led to faster categorization of the subsequent targets, as compared to scrambled primes (mean RT for identical primes = 606 ms, *SE* = 25 ms vs. scrambled primes = 630 ms, *SE* = 27 ms; Bf-*p* < 0.01). However, both these effects should be interpreted with caution, because there was also a significant interaction between *group, prime location*, and *prime condition* [*F*_(1, 16)_ = 6.26, *p* < 0.05, _*p*_η^2^ = 0.28] which clarified their nature. Namely, the findings were such that only participants in the LVF-A group responded significantly faster when primes presented in their dominant VHF were identical rather than scrambled (mean RT for identical primes = 629 ms, *SE* = 35 ms vs. scrambled primes = 659 ms, *SE* = 38 ms; Bf-*p* < 0.01). In the RVF-A group, this effect missed the significance threshold (mean RT for identical primes = 578 ms, *SE* = 40 ms vs. scrambled primes = 598 ms, *SE* = 37 ms; Bf-*p* = 0.07). Nevertheless, the impact of right-sided identical priming in the RVF-A group was revealed by a planned *a priori t*-test [*t*_(8)_ = 2.47, *p* < 0.05]. These effects are shown in Figure 4. Finally, the most important significant interaction was revealed between *group, target category*, and *prime condition* [*F*_(1, 16)_ = 4.47, *p* = 0.05, _*p*_η^2^ = 0.22]. Participants who were classified as the LVF-A group responded faster when non-tools were preceded by identical compared to scrambled primes (mean RT for identical primes = 635 ms, *SE* = 38 ms vs. scrambled primes = 657 ms, *SE* = 36 ms; Bf-*p* < 0.01). RVF-A group, on the other hand, showed a clear trend toward faster categorization of tools when they were preceded by identical compared to scrambled primes (mean RT for identical primes = 562 ms, *SE* = 40 ms vs. scrambled primes = 587 ms, *SE* = 41 ms; Bf-*p* = 0.06). Indeed, this effect was significant as shown by a planned *a priori t*-test [*t*_(8)_ = 2.80, *p* < 0.05]. These results are shown in Figure 5. The mean RTs and average accuracy for all the conditions are listed in Table 2.

**Table 2:** **Primes in VHFs—Experiment 2**.

**Trial type**			**Response time (ms)**	**St. error**	**Accuracy (%)**	**St. error**	***N***
LVF	Tool	Identical prime	601	28	89.1	2.0	18
		Scrambled prime	619	29	88.6	1.8	18
	Non-tool	Identical prime	611	24	95.7	1.0	18
		Scrambled prime	641	27	96.0	1.0	18
RVF	Tool	Identical prime	606	31	89.4	1.7	18
		Scrambled prime	622	30	87.4	2.0	18
	Non-tool	Identical prime	624	29	95.7	0.9	18
		Scrambled prime	629	24	96.0	1.0	18

*Target location (Left Visual Field, LVF; Right Visual Field, RVF), target category (tool, non-tool), prime condition (identical, scrambled) with their mean response times (ms), accuracy (%), and their standard errors of the means, for Experiment 2 with primes presented in either LVF or RVF are listed*.

### Discussion of experiment 2

Because the task in Experiment 2 involved a centrally presented target stimulus (encoded by both hemispheres), whose processing could potentially be affected by the laterally presented primes, the results obtained with this paradigm may tell us substantially less about the laterality of neural mechanisms involved in the visual categorization of man-made objects, but can potentially tell us much more about the inner organization of the processes that subserve this function.

Despite a very complex pattern of results obtained in this experiment, two patterns of outcomes are clear-cut. No surprisingly, the *typical* (RVF-A) group responded faster following identical priming coming from its dominant right visual field, and the *atypical* (LVF-A) group responded faster following identical priming coming from its dominant left visual field. Yet, this was only the case when tools and non-tools were collapsed. In sharp contrast, as the most crucial outcome of our study indicates, whereas the effect of greater facilitation of RTs following identical priming in the *typical* group was driven primarily by faster reaction times to tools, the greater facilitation of RTs following identical priming in the *atypical* group was driven primarily (indeed, almost exclusively) by faster reaction times to non-tool targets. It must be clearly emphasized, though, that the latter two effects were now independent of the side in which the priming stimulus occurred.

The results of Experiment 2 therefore suggest that in the case of cognitive mechanisms that are strongly *encapsulated* (i.e., form a relatively independent module specialized for certain kind of stimulus encoding, Króliczak et al., 2012; cf. Clark, 2009), the presence of the prime in the information-processing stream will affect the subsequent (centrally-categorized) target irrespective of priming side and regardless of which hemisphere is involved more in the categorization process itself. This is at least the case in the LVF-A group, where the categorization of non-tools was in fact facilitated (in terms of RTs) by identical primes irrespective of their location. Such a pattern of performance also implies that at least in individuals with atypically lateralized object encoding, and putatively atypical organization of other cognitive skills, (1) the concepts of man-made objects which do not have close affinity to any specific representations of manual dexterity are still organized more symmetrically (see also Experiment 1, e.g., Ishai et al., 1999, 2000; Verma and Brysbaert, 2011), but despite being distributed across the two hemispheres, and somewhat counterintuitively, (2) the critical mechanisms for human artifact categorization seem to be more specialized (encapsulated) for non-tool objects than for manipulable tools (whose usage requires a proper grasp and sequence of hand movements). Indeed, the presence of such a specialized mechanism may be responsible for more accurate categorization of non-tools in this particular group. Conversely, in the RVF-A group, a faster categorization of tools preceded by primes on any side implies a more specialized mechanisms contributing to the processing of this narrower category of objects, which is in line with neuropsychological and neuroimaging evidence from right-handed (most of the time having typical organization of cognitive skills) subjects (for a review, see Frey, 2008).

This study clearly shows that cognitive decisions involving different categories of objects can be easily primed (e.g., Garofeanu et al., 2004; Garcea et al., 2012). Yet, the strength and direction of the effect will depend on object category—will be different for non-tools and tools—and on the mechanisms predominantly involved in their processing. E.g., the priming effect for non-tools may depend more on the overall target shape, whereas for tools, on its afforded action features, i.e., its graspability. Indeed, we expect that the priming effects would be different not only for disparate object categories, but also for the type of task to be performed, including both perceptual and action decisions (e.g., Helbig et al., 2006; McNair and Harris, 2012; Bub et al., 2013; cf. Craighero et al., 1996; Króliczak et al., 2006).

## General comments

The way the representations of man-made objects are organized and/or lateralized in the human brain, and as a consequence the efficiency with which they are utilized in cognitive processes is likely to depend on—among other factors such as the strength of connections between the object concept and its relevant functional properties, or the distance (the number of levels/nodes/synapses) separating such conceptual and functional knowledge—whether or not a particular type of object affordances is critically linked to the representations of manual skills (e.g., Bub et al., 2008; Pellicano et al., 2010; Proverbio et al., 2011). For example, the chair can be effectively moved closer to the body (or rather legs) with the hands but what it affords has nothing to do with skilled hand movements (thus representing a low degree of manipulability). This is probably why the concepts of tools are special: a reason being the gradually acquired privileged link between the functional knowledge and the knowledge of the relevant movements (i.e., manual dexterity) that comes into play with deft tool use. Such representations and/or links between them are clearly absent in kids who can already name tools but cannot effectively use them, not to mention pantomiming their use (O'Reilly, 1995; Landau et al., 1998).

In right-handers, most of the mechanisms underlying tool categorization and/or tool use abilities, as well as the processes that enable orchestrated interactions of the disparate and often differently localized mechanisms involved in dealing with this subcategory of *human artifacts*, are lateralized to the left hemisphere. Of course, things may change substantially when a preference for using the left hand gets factored in the build-up of their representations. Hence, in some left-handed individuals tool concepts seem to have greater affinity to the right-hemisphere mechanisms underlying hand dominance, although, as our Experiment 1 shows, in the majority of sinistrals this is not the case (cf. Króliczak et al., 2011; Vingerhoets et al., 2013). If the former happens, though, this does not necessarily entail that the representations of other man-made objects are automatically reorganized, shifted and/or moved to the opposite (left) hemisphere (as clearly demonstrated by Experiment 2). Indeed, the mechanisms invoked during interactions with non-tools may in such cases depend further on the more distributed, bilateral processing, being at the same time less prone to local one-sided injuries.

If we assume that tool concepts form only a unique subset of the category of man-made objects including non-tools, or there is a substantial overlap between the two categories, then a very counterintuitive idea emerges. Indeed, this idea is of paramount importance for the neurocognitive rehabilitation of apraxia (cf. Oliveira and Brito, 2014). Namely, this study suggests that in patients with atypically organized skills the most effective way of alleviating tool-related conceptual and/or motor deficits that would follow right-hemisphere damages might be targeting first their relatively preserved skills to deal with non-tools. After all, as we demonstrated, some of the ***processes*** involved in the categorization of non-tools (see Experiment 2) are in such individuals organized quite similarly to the ***mechanisms*** invoked directly during the categorization of tools (see Experiment 1).

This study as a whole convincingly shows that individuals with atypically organized cognitive skills are not just mirror reversed images of *typical* subjects (cf. Lewis et al., 2006). This is particularly true about the way the representations of tools are encoded and retrieved in the *atypical* brain. Notably, although an objective method was used here to divide participants into groups (which happened to be equal) with typical and atypical laterality of object categorization, this should not be construed as evidence that 50% of our left-handers demonstrated atypical laterality of tool processing. Depending on how this issue is approached, e.g., based exclusively on Experiment 1 or Experiment 2, only 38.9% or just 33.3% of sinistrals, respectively, demonstrated the atypical left-visual field (right hemisphere) advantage for tool categorization (consistent with Króliczak et al., 2011).

Based on both experiments, there is evidence to indicate that the atypical group seems to possess more refined representations of non-tool objects, despite the involvement of both hemispheres in the processing of such human artifacts. In contrast, individuals with typically organized brains possess more fine-grained representations of tools whereas the non-tool category seems more diffused. Indeed, in our opinion, equivocal effects that were likely obtained while testing left-handers are to blame for the exclusion of sinistrals from scientific research and the lack of interesting reports on their cognitive skills (see also Willems et al., 2014).

## Limitations of the study

It would be of great interest to test whether or not individuals with atypically organized tool processing would also demonstrate atypical (i.e., bilateral or right-sided) organization of language skills. This could have been easily tested using the VHF paradigm as shown by Hunter and Brysbaert (2008). Based on Króliczak et al. (2011), we expect that no more than 25% of these participants would show atypical language laterality.

In the context of Experiment 1, it would be desirable to include a third type of distracter, i.e., a neutral one, in order to further investigate the possible facilitation or interference effects. In Experiment 2, on the other hand, the inclusion of incongruent primes (i.e., representing objects from the other category) could shed some new light on the efficiency and perhaps the more detailed organization of mechanisms and processes involved in the categorization of man-made artifacts.

## Conclusions

Although dextrals were not included in this project, the results we obtained clearly suggest that dividing study participants based on hand dominance, not to mention the exclusion of sinistrals, makes no sense. A much more reasonable approach would be to group subjects into those representing typical and atypical laterality of cognitive skills. Such a change in the recruitment, inclusion, and assignment process could in fact lead to new and hopefully more adequate models of the organization of functions in the healthy brain, which in turn could generate new approaches to neurocognitive rehabilitation. By the same token, these results also indicate that collapsing across all left-handed individuals in fMRI analyses might not be the most advisable strategy.

## Author contributions

This project was conceptualized by BM and GK. Data was collected by BM, and analyzed by BM and GK. The manuscript was written by GK and BM.

### Conflict of interest statement

The authors declare that the research was conducted in the absence of any commercial or financial relationships that could be construed as a potential conflict of interest.

## Appendix 1

### Stimuli used in experiment 1

*Tools:* ax, baseball bat, broom, brush, comb, fork, hammer, iron, key, knife, ladle, lipstick, nail file, net, paintbrush, pen, pliers, rake, rolling pin, ruler, saw, scissors, screwdriver, spoon, stethoscope, syringe, tennis racket, toothbrush, watering can, wrench.

*Non-tools:* accordion, airplane, anchor, belt, bottle, candle, cigarette, cutting board, dart, envelope, football, glasses, guitar, hanger, kite, ladder, lamp, light bulb, nail, padlock, pipe, plug, roller-skate, screw, shoe, sock, tie, trumpet, violin, watch.

### Stimuli used in experiment 2

*Tools:* bottle opener, brush, cigarette lighter, comb, corkscrew, drill, food mixer, fork, hammer, iron, key, knife, mouse, paint roller, paintbrush, pen, pincers, pliers, punch, razor, saw, scissors, screwdriver, snap-off knife, spatula, spoon, tenderizer, thimble, toothbrush, wrench.

*Non-tools:* basket, battery, belt, book, bottle, charger, compact disc, cover, dumbbell, extension cord, frame, glasses, glove, hanger, hat, headphones, knight, light bulb, mascot, necklace, padlock, pillow, shoe, sock, spool, suitcase, toilet roll, toothpaste tube, USB flash drive, watch.
